# Establishment and Clinical Applications of a Portable System for Capturing Influenza Viruses Released through Coughing

**DOI:** 10.1371/journal.pone.0103560

**Published:** 2014-08-01

**Authors:** Etsuko Hatagishi, Michiko Okamoto, Suguru Ohmiya, Hisakazu Yano, Toru Hori, Wakana Saito, Hiroshi Miki, Yasushi Suzuki, Reiko Saito, Taro Yamamoto, Makoto Shoji, Yoshihisa Morisaki, Soichiro Sakata, Hidekazu Nishimura

**Affiliations:** 1 Virus Research Center, Clinical Research Division, Sendai Medical Center, Sendai, Japan; 2 Department of International Health, Nagasaki University Graduate School of Biomedical Sciences, Nagasaki, Japan; 3 Department of Respiratory Medicine, Sendai Medical Center, Sendai, Japan; 4 Division of Public Health, Department of Infectious Disease Control and International Medicine, Graduate School of Medical and Dental Sciences, Niigata University, Niigata, Japan; 5 Department of International Health, Institute of Tropical Medicine, Nagasaki University, Nagasaki, Japan; 6 Shoji Clinic, Sendai, Japan; 7 Japan Self-Defense Force Sendai Hospital, Sendai, Japan; 8 Takasago Thermal Engineering Co., Ltd., Tokyo, Japan; The University of Hong Kong, Hong Kong

## Abstract

Coughing plays an important role in influenza transmission; however, there is insufficient information regarding the viral load in cough because of the lack of convenient and reliable collection methods. We developed a portable airborne particle-collection system to measure the viral load; it is equipped with an air sampler to draw air and pass it through a gelatin membrane filter connected to a cone-shaped, megaphone-like device to guide the cough airflow to the membrane. The membrane was dissolved in a medium, and the viral load was measured using quantitative real-time reverse transcriptase-polymerase chain reaction and a plaque assay. The approximate viral recovery rate of this system was 10% in simulation experiments to collect and quantify the viral particles aerosolized by a nebulizer. Using this system, cough samples were collected from 56 influenza A patients. The total viral detection rate was 41% (23/56), and the viral loads varied significantly (from <10, less than the detection limit, to 2240 viral gene copies/cough). Viable viruses were detected from 3 samples with ≤18 plaque forming units per cough sample. The virus detection rates were similar among different groups of patients infected with different viral subtypes and during different influenza seasons. Among patients who did not receive antiviral treatment, viruses were detected in one of six cases in the vaccinated group and four of six cases in the unvaccinated group. We found cases with high viral titers in throat swabs or oral secretions but very low or undetectable in coughs and vice versa suggesting other possible anatomical sites where the viruses might be mixed into the cough. Our system is easy to operate, appropriate for bedside use, and is useful for comparing the viral load in cough samples from influenza patients under various conditions and settings. However, further large-scale studies are warranted to validate our results.

## Introduction

Coughing plays an important role in the rapid spread of influenza infections among humans. Influenza virus-borne bio-particles are discharged from an infected person through coughing and transmitted to uninfected person(s). However, whether influenza virus particles are directly transmitted to the target host as large droplets or through inhalation by the host as small airborne particles remains controversial.

For example, Brankston et al. and Tellier reviewed many clinical and epidemiological studies, as well as experimental transmission studies using animal models and volunteers, and drew strikingly different conclusions: Brankston et al. did not support the airborne route [1], whereas Tellier advocated that the importance of the airborne route in the natural transmission of influenza infections [2], [3]. To address these controversies, Wong et al. [4] conducted a spatiotemporal analysis during an influenza outbreak in a hospital ward and reported that infections spread along the direction of airflow from the index case. Furthermore, Lindsley et al. [5] and Blachere et al. [6] detected the airborne virus in healthcare facilities treating influenza patients. Thus, it is important to assess airborne transmission to discern the amount of virus released into environmental air through coughing.

Recently, viral RNA was detected in voluntary cough samples of influenza patients by many researchers using their own methods. For example, Stelzer-Braid et al. [7] used a continuous positive airway pressure mask with a collection disk composed of a dielectric material, and Lindsley et al. [8] used a biosampler (SKC Inc., Eighty Four, PA, USA) and a two-stage bioaerosol cyclone sampler (National Institute for Occupational Safety and Health, Pittsburgh, PA, USA). Moreover, Bischoff et al. [9] collected viruses by sampling room air for >20 min using the Andersen impaction air sampler (Thermo Fisher Scientific, Waltham, MA, USA) placed close to the patient, and Milton et al. [10] used self-produced G-II sampler designed for collection of the exhaled breath and cough. However, the systems used in the aforementioned studies did not appear to be optimal for collecting samples from many subjects in medical settings or common areas, because of issues of burden to patients, portability and simplicity.

**Figure 1:**
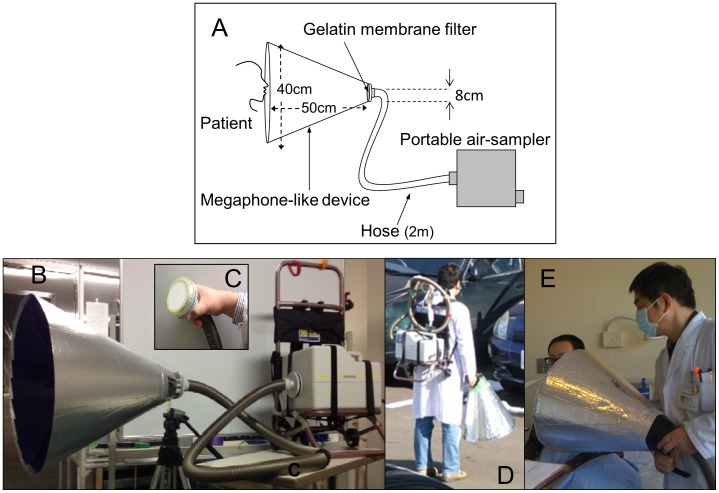
The system cough collection developed in this study. A schematic diagram of the total system (A). A picture of the composition: the pump, hose, and cone shaped, megaphone-like device, from the right (B). The gelatin membrane filter (C) was attached to end of the hose and connected to the tip of the cone to entrap the particles passing in the airflow. The complete system is portable, even on ones back (D), and can be easily used in many situations, especially at bedside (E).

In the present study, we developed a portable system to easily collect bio-particles released by coughing at bedside within a short time without burdening the patients (Figure 1), and quantified the viral load with a definite recovery rate. The methodology and collection results from influenza cases under various conditions, such as status of vaccination and antiviral treatment, as well as possible applications for analyses of the effects of these interventions are discussed. Furthermore, combining the viral load data of the cough samples with those of throat swabs and oral secretions, we propose some interesting possibilities regarding the anatomical sites where the viruses might be mixed with a cough.

## Materials and Methods

### Ethics Statement

This study was approved by the ethical committee of Sendai Medical Center (Sendai City, Miyagi Prefecture, Japan), and written informed consent was obtained from all patients.

### Device for collection of bio-particles

Airborne particles in coughs and mists, generated in laboratory experiments to simulate coughs, were collected using an airborne particle-sampling unit composed of portable air sampler (MD8 AirScan Sartorius AG, Göttingen, Germany) connected by a flexible polyvinyl chloride hose with reinforced ends (#17085; inner diameter, 32 mm; length, 2 m; Sartorius AG) (Figure 1A) to a gelatin membrane filter (#12602-080-ALK; diameter, 8.0 cm; pore size, 3.0 µm; Sartorius AG) (Figure 1 A, C) and equipped with a cone-shaped, megaphone-like device (length, 50 cm; entrance diameter, 40 cm) made of a polycarbonate resin sheet (Figure 1 A, B) to guide the airflow to the membrane. The outer surface of the device was coated with aluminum foil to reduce the static electric charge of the inner surface.

### Collection of clinical samples

Cough, throat swab and oral samples were collected, in this order, from individuals exhibiting symptoms of influenza who visited one of three medical facilities in Sendai City, Japan. Among them, those who received final diagnosis of influenza by viral isolation from throat swab samples were enrolled in this study. Samples from 16 patients from Sendai Medical Center (average age, 32.4 ± 16.6 years), 39 from Japan Self-Defense Force Sendai Hospital (average age, 22.1 ± 3.2 years), and 1 from Shoji Clinic (age, 44 years) were collected from January 2008 to February 2011 and included for analysis.

For sample collection, patients were asked to cough voluntarily approximately 20 times at their own pace and without great effort into the cone entrance toward the gelatin membrane filter, which was at the opposing tip of the cone. The connected sampler was set at a constant airflow rate of 8.0 m^3^/h, which is the maximum pumping rate according to the manufacturer and was previously adopted in a swine influenza study [11]. The pumping duration was from 1 min before the start of coughing and continued until at least 1 min after the last cough. The filter to trap the bio-particles was cooled immediately after collection until it was transported to the laboratory where it was then dissolved in 10 mL of Eagle's minimum essential medium (MEM; Sigma-Aldrich, St. Louis, MO, USA) supplemented with 50 U of penicillin and 50 µg/mL of streptomycin at 37°C. The samples were aliquoted and then assessed using a plaque assay, whereas the remaining samples were stored at −80°C until quantification of the viral gene copy number. Throat swabs were collected from the pharyngeal walls exclusively by one of the investigators (E.H.) to avoid technical variation in sampling techniques by multiple collectors using cotton swabs. Then, the swabs were vigorously washed in MEM transport medium supplemented with 50 U of penicillin and 50 µg/mL of streptomycin. The oral secretions consisted mainly of saliva and mucus produced by the oral membrane and/or mucus brought by a cough from the deeper respiratory tract was spat by patients in Petri dishes.

### Virus and devices for airborne experiments

Influenza virus A/Aichi/2/68 (H3N2) was propagated in the allantoic cavities of fertilized chicken eggs. The allantoic fluid containing the viruses was harvested and atomized in simulation experiments using an electric compressor nebulizer (NE-C16; Omron, Kyoto, Japan).

### Concentration and quantification of the viruses eluted in the gelatin solution

The gelatin membrane was dissolved in 10 mL of MEM and treated with 10 µg/mL of collagenase (Collagenase S-1, Nitta Gelatin, Osaka, Japan) at 37°C for 1 h and ultra-centrifuged at 125,000 g for 100 min to obtain viral precipitate, which was then dissolved in extraction buffer included with the RNA extraction kit (QIAamp Viral RNA Mini Kit, Qiagen, Valencia, CA, USA); the viral RNA was extracted in 80 µL of RNAse-free water according to the manufacturer's instructions. The RNA solution was concentrated using a freeze-drying system composed of a freeze dryer (FD-80; Eyela; Tokyo Rikakikai Co., Ltd., Tokyo, Japan), a centrifugal concentrator (VC-12S; TAITEC, Saitama, Japan), and a vacuum pump (MFG; Hitachi, Tokyo, Japan). The total amount of extracted RNA was used for complementary DNA (cDNA) synthesis using the reverse transcription kit (High-Capacity cDNA Reverse Transcription Kit, Applied Biosystems, Foster City, CA, USA) with random primers following the manufacturer's protocol. The amount of generated cDNA was measured with quantitative real-time reverse transcriptase-polymerase chain reaction (qRT-PCR) using the MiniOpticon system with CFX Manager software (Bio-Rad Laboratories, Hercules, CA, USA), influenza A matrix protein 1 (M1)-specific primers, and probes designed by Daum et al. [12]. All reactions were performed in 48-well plates in duplicate or triplicate. A standard curve was generated from 10-fold serial dilutions of the RNA from the M1 cDNA of influenza virus strain A/Aichi/2/68. Negative controls without templates were included in each plate.

### Viral isolation and plaque assay

Throat swab samples in MEM transport medium were centrifuged at low-speed (1500 g) for 15 min, and then inoculated onto Madin-Darby canine kidney (MDCK) cells for viral culture [13]. Viral isolation was assessed within a week by identifying the specific cytopathic effect. The dissolved membrane specimens containing viruses of the coughs were the serially diluted 10-fold with MEM and inoculated onto MDCK cells. The diluted specimens were assessed for active viruses using a conventional plaque assay [14] within 1 h after collection.

## Results

### System establishment for viral collection and quantification

The collection system was equipped with a portable air sampler unit containing a gelatin membrane filter and a large cone-shaped, megaphone-like device to guide the expelled particles to the filter (Figure 1 A, B). The length and maximum diameter of the cone were 50 and 40 cm, respectively, which was based on information on cough airflow provided by *schlieren* (German word, indicating optical inhomogeneities) analysis, as per the report by Tang et al. [15] and our recent study of vector analysis [16]. These two reports estimated the range of the momentum of coughs to be approximately 30 cm or less from the participant's mouth and thereafter, the particles spread by diffusion. Considering this assumption, the length of the device was determined as 50 cm to include possible outlier cases. The particle velocity by coughing should slow down at the filter to at least less than that of the suctioning airflow (0.56 m/s, theoretically calculated).

To evaluate the efficiency of our system to measure the total viral load in a cough, we attempted to separately estimate the rough recovery rates of the collection and quantification processes.

**Figure 2:**
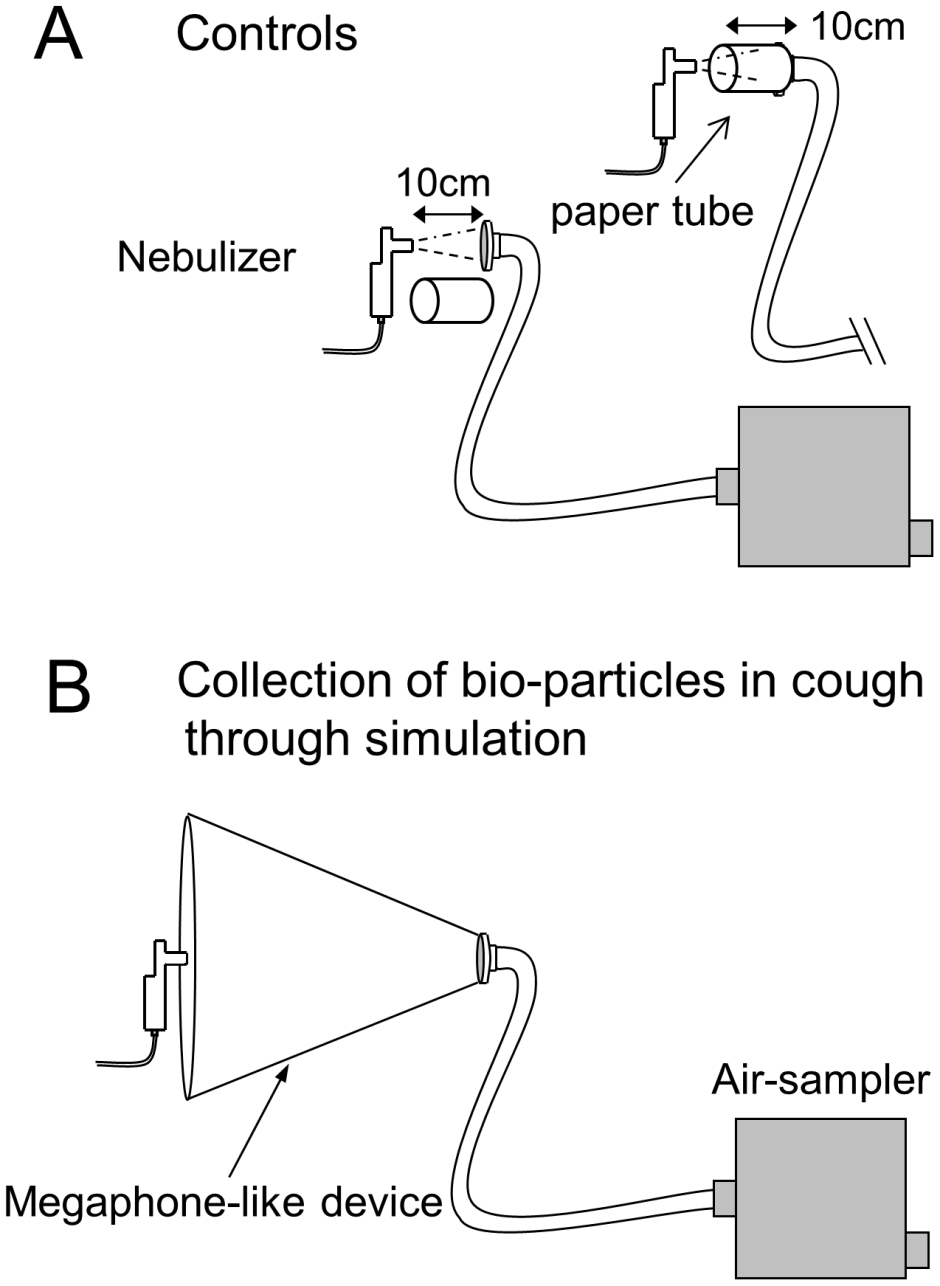
A schematic diagram of simulation experiments to collect biomist particles. Settings for the experiments to simulate biomist collection from coughs to estimate the viral recovery rate of this system. Control experiments with mist particles generated from a nebulizer were drawn directly into the gelatin filter membrane, with or without a hollow paper tube to guide the mist to the membrane (A), and the collection of the particles through the cone-shaped device to guide particles expelled through a cough (B).

For the collection process, experiments to simulate human coughing were performed using a nebulizer. Size distribution patterns of the particles were confirmed to be mostly resembled between the mist of the nebulizer and human coughs (Figure S1). First, the viral fluid containing approximately 10^8^ plaque forming units (PFU)/mL of influenza virus was atomized by a nebulizer every 0.5 s for 3 s, which was repeated 10 times, and directed toward a gelatin membrane filter set 10 cm from the nebulizer. The generated viral mist was drawn directly into the gelatin filter membrane apparently by robust pumping of the sampler, which was confirmed against a black paper background and was visible by the naked eye. A similar experiment was repeated using a hollow paper tube (length, 10 cm; inner diameter, 8 cm) to guide the mist to the membrane, of which the outer and inner surfaces were coated with aluminum foil to prevent static charge (Figure 2 A). This tube was placed to minimize the possibility that the collection of mist by the membrane might be drastically reduced by the diffusion of the mist, and the result was compared with that of an experiment conducted without the use of a tube. Then, the cone-shaped device employed for cough collection was attached, the nebulizer was placed at the entrance of the device, and the viral mist collection experiments were repeated. (Figure 2 B). We observed that the generated mist was smoothly sucked into the cone-shaped device. After mist collection, the gelatin membrane was dissolved in MEM and the viral M1 gene copy-number was quantified using qRT-PCR. The results showed that the recovery rates of the viruses between the controls with and without the tube were almost equal and the results of the system employing the cone-shaped device were approximately 58% of the controls. We obtained similar results when less concentrated viral fluid (10^4^ PFU/mL) was atomized (Table 1).

**Table 1:** Viral recovery rate of collection and quantification system.

The recovery rate in collection system using a megaphone-like device					
Setting for collection of the mist		Experiment 1 (high dose)^a^		Experiment 2 (low dose)^b^	
		Viral load^c^ (copies)	Recovery rate^d^ (%)	Viral load (copies)	Recovery rate (%)
Controls: close to membrane^e^	with hollow paper tube	3.08×10^7^ (SE 1.20×10^7^)	100	NT	NT
	without the tube	3.11×10^7^ (SE 2.63×10^6^)	100	5.9×10^3^	100
With a megaphone-like device^f^		1.80×10^7^ (SE 1.72×10^6^)	58	3.2×10^3^	54
**The viral recovery rate in quantification process**					
Steps in the quantification system		Experiment 3 (high dose)		Experiment 4 (low dose)	
		Viral load (copies)	Recovery rate^g^ (%)	Viral load (copies)	Recovery rate (%)
Starting materials^h^		4.4×10^7^	100	1.9×10^5^	100
Membrane dissolution^i^		3.6×10^7^	82	1.6×10^5^	84
Collagenase treatment^j^		4.0×10^7^	91	1.9×10^5^	100
Concentration^k^		9.4×10^6^	21	3.6×10^4^	19

a The experiments were performed in triplicate for the high amount experiment controls and the number is the average and standard error (SE) of the results.

b The low dose experiment was performed once to confirm the reproducibility of the recovery rate, as determined by the high dose experiment.

c Average copy number of the influenza A M1 gene in the gelatin membrane.

d Percentage of viral load on the gelatin membrane to that of collection when the nebulizer was set close to the membrane.

e The nebulizer was set 10 cm from the gelatin membrane filter through which the viral mist was suctioned.

f The cone-shaped, megaphone-like device of 50 cm in length was connected with the gelatin membrane filter and the nebulizer was set at the entrance of the device.

g Percentage of viral load in the product of each step to those at the start of each step.

h Viral load in the viral fluid poured into the gelatin membrane solution.

i Viral load in the solution of the gelatin membrane filter dissolved in 10 mL of MEM.

j The gelatin solution containing the virus was digested with 10 µg/mL of collagenase.

k The digested fluid was ultra-centrifuged at 125,000 g for 100 min and the viral RNA was extracted from the precipitate and concentrated by freeze-drying and vacuum centrifugation.

To estimate the recovery rate of the quantification process, we measured the amount of viral RNA in each step (Table 1). Briefly, 10 µL of viral fluid was poured into a gelatinous solution composed of the membrane dissolved in 10 mL of MEM. The solution was treated with collagenase to digest the gelatin to prevent regelatinization and diminish the viscosity to ultimately improve the efficiency of viral condensation in the subsequent ultracentrifugation step. The viral precipitate obtained with ultracentrifugation was subjected to RNA extraction and concentrated by freeze-drying and vacuum-centrifugation. Our results showed that the subtotal recovery rate of the quantification process was estimated at about 20%, and the total recovery rate of our system was ≥10%.

### Quantification of viral loads in coughs of influenza patients

To apply the above system in actual clinical setting, we collected particles from coughs of 56 influenza patients, whose diagnosis was confirmed by viral isolation from throat swabs. The average patient age was 25.4 ± 10.5 years (range, 5–67 years), 88% of the samples were from 18–38-year-old patients, and the male-to-female ratio was 3∶1. None of the patients had significant health problems. Forty-five (80%) cases were infected with influenza A(H1) virus, 5 (9%) with A(H3), and 6 (11%) with A(H1N1)pdm09 (Table S1).

Next, the amount of viable viral particles trapped by the gelatin membrane was quantified using a conventional plaque assay. Viable viruses were detected in 3 (5%) samples with titers of 1.5, 1.5, and 18 PFU per cough of viral subtypes A(H1), A(H3) and A(H1N1)pdm09, respectively. Due to the potential existence of non-viable virions, qRT-PCR targeting a viral gene was simultaneously performed. The detection limit for reliable quantification in our qRT-PCR system was preliminary determined as 10 gene copies per µL cDNA of the influenza virus gene. Calculations with dilution rates by the volume of the membrane solution and the times of coughs, as well as results of our actual cases, determined that the detection limit was about 10 copies per cough. Using this detection limit as a parameter, the cases were deemed as either positive or negative for the presence of influenza virus. Of the 23 (41%) cases determined as positive, and the viral load varied widely from 10 to 2240 copies/cough. Seventeen (30%) samples contained a few to several dozen copies/cough, five (9%) contained 100–200 copies/cough, and one contained an exceptionally high level, i.e., 2240 copies/cough; the remaining samples had an average and median of 63 and 44 copies/cough, respectively, with a standard deviation of 56. The highest viral content was found in a sample obtained at day 3 of illness in a patient with the influenza A(H1N1)pdm09 virus infection, which was one of three cases in this study in whom active virus was detected (the plaque count  =  18/cough).

**Figure 3:**
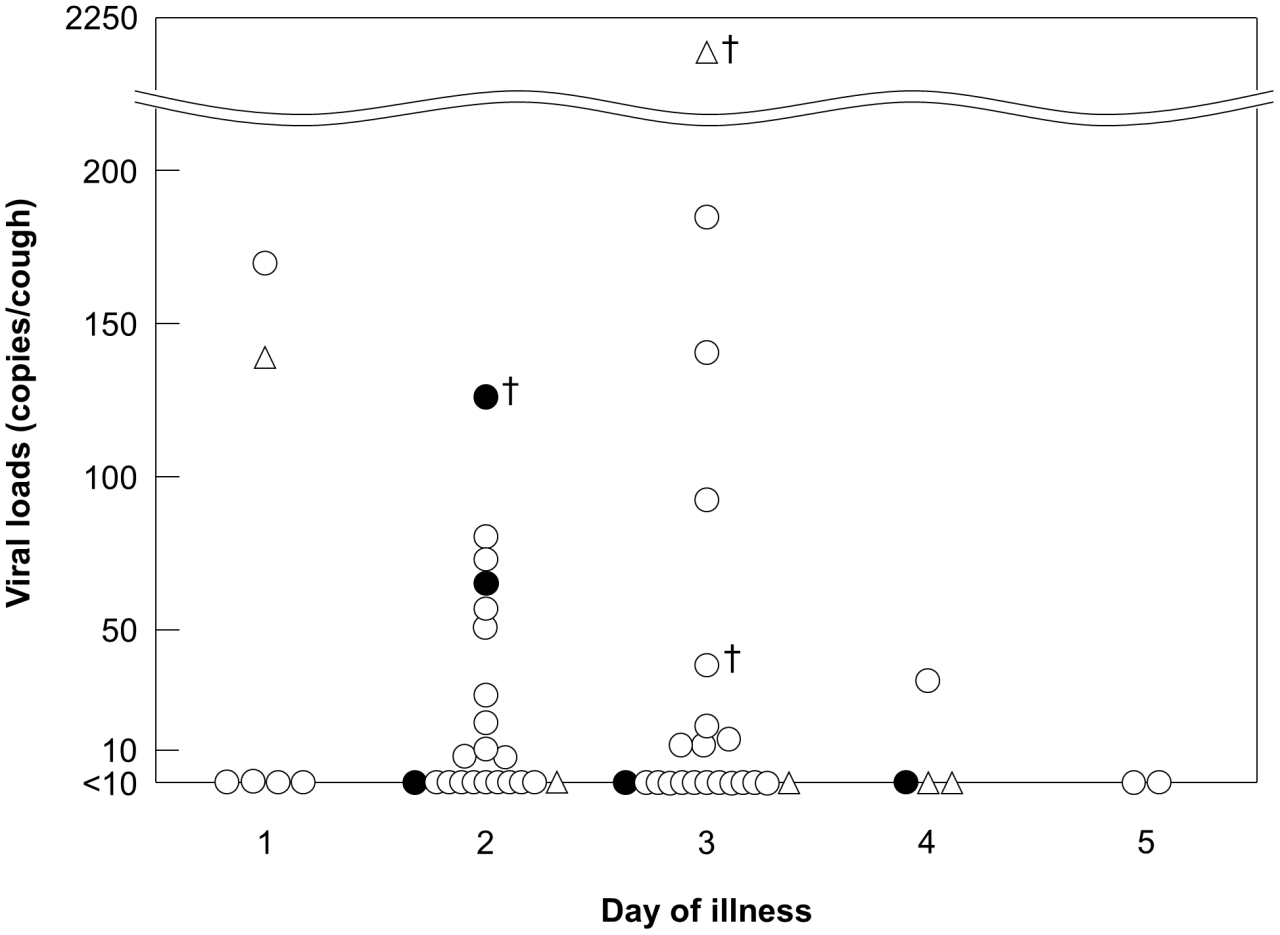
Viral loads in coughs of influenza patients. Cough samples were collected from individual patients and subjected to viral quantification. The viral load/cough was plotted on the ordinate along with days of illness during sample collection on the abscissa. The onset day of any influenza symptom was defined as day 1. Viral subtypes are presented using different symbols: open circle, closed circle, and open triangle represent infections with A(H1), A(H3), and A(H1N1)pdm09 virus, respectively. Daggers indicate cases in which the active virus was detected using a conventional plaque assay.

The results were analyzed from different perspectives depending on the characteristics of individual cases and initially graphed with the days of illness along the abscissa. Day 1 was defined as the onset day of any influenza symptom (e.g., high fever and coughing). We did not find any significant differences in the viral detection rates among groups of viral subtypes (Figure 3, Table S1), influenza seasons (Table S1, Figure S2), or gender (Table S1). Moreover, we did not find any specific tendency related to patient body temperature (Figure S3).

### Viral loads in coughs from patients following vaccination and/or antiviral treatment

For infection control, it is important to determine the presence or absence of vaccination and antiviral treatment effects on viral load. Therefore, we analyzed the obtained data to assess the impact of patient vaccination and antiviral treatment status on the viral load in a cough. Among 56 cases, 11 (20%) received influenza vaccinations before the influenza season and 34 (61%) received antiviral treatments with the neuraminidase (NA) inhibitors oseltamivir or zanamivir at the time of cough collection.

**Figure 4:**
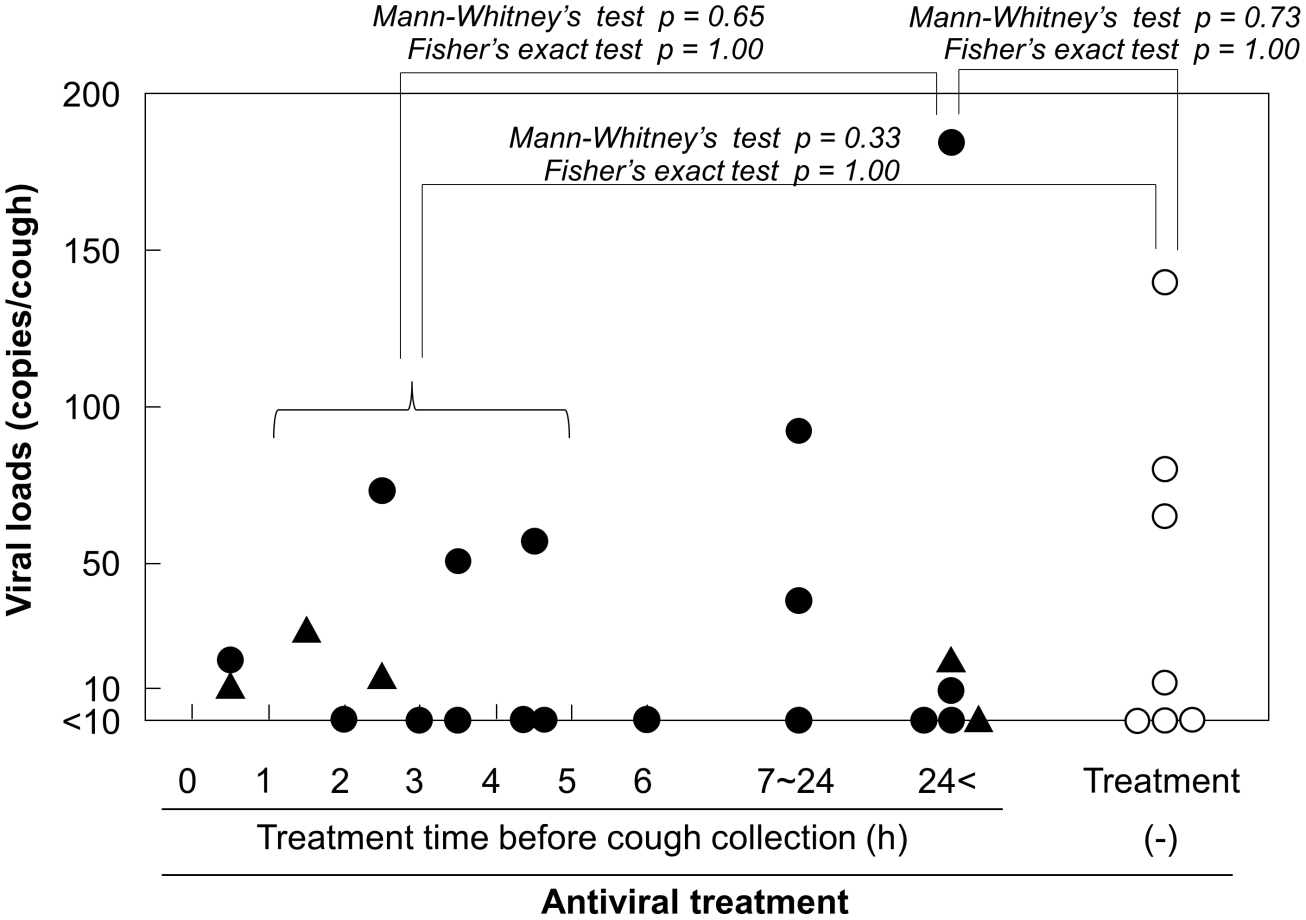
Viral loads in coughs of unvaccinated influenza patients. Data from days 2 and 3 of symptom onset were plotted together in respect to the status of receiving antiviral treatment, including treatment time before cough collection. The open circle, closed circle, and closed triangle represent subjects untreated, and treated with oseltamivir or zanamivir, respectively. Viruses isolated in oseltamivir-treated cases were all subtype A(H1), which is considered to be oseltamivir-resistant.

First, we graphed the viral loads in individual cases along with the state of antiviral treatment against time from treatment initiation to cough collection (Figure 4). Here we plotted the data of days 2 and 3 together because there was no clustering of vaccination or antiviral treatment status among cases over these days.

For cases treated during a supposedly sufficient time for viral replication before cough collection (>24 h) in the host, the detection rate was similar among control untreated cases (3/6, 50% vs 4/7, 57%) as was the viral load (p = 1.00, Fisher's exact test; p = 0.73, Mann–Whitney U test). We were also interested in the results of patients with a shorter duration of treatment (1.5–4.5 h) from the perspective of whether viral release from the respiratory tract by coughing could be inhibited by drug administration after the viral budding process concluded. This point of view was based on the mechanisms of the action of NA inhibitor drugs, as the timing of this action is considered important at the moment of viral release at viral budding sites to inhibit release by the inactivation of the viral NA enzyme. Thus, we wished to determine whether there was another mechanism by which the drugs enhanced rebinding of the virus to the cell surface, which had already been released from the cell surface, and thus reduce the viral load of the cough or not. We, therefore, added the results of 10 such cases to the same graph and found that the virus detection rate of these cases (5/10, 50%) was also similar to those of both untreated and sufficiently treated cases (both p = 1.00, Fisher's exact test), as was the viral load (p = 0.33 and 0.65, Mann–Whitney U test, respectively). However, when the medians were compared, these short-term treated cases showed no significant difference with the untreated cases, but the sufficiently treated cases showed apparently smaller viral loads (median  = 50.6, 72.8, and 19.7 particles/cough, respectively).

**Figure 5:**
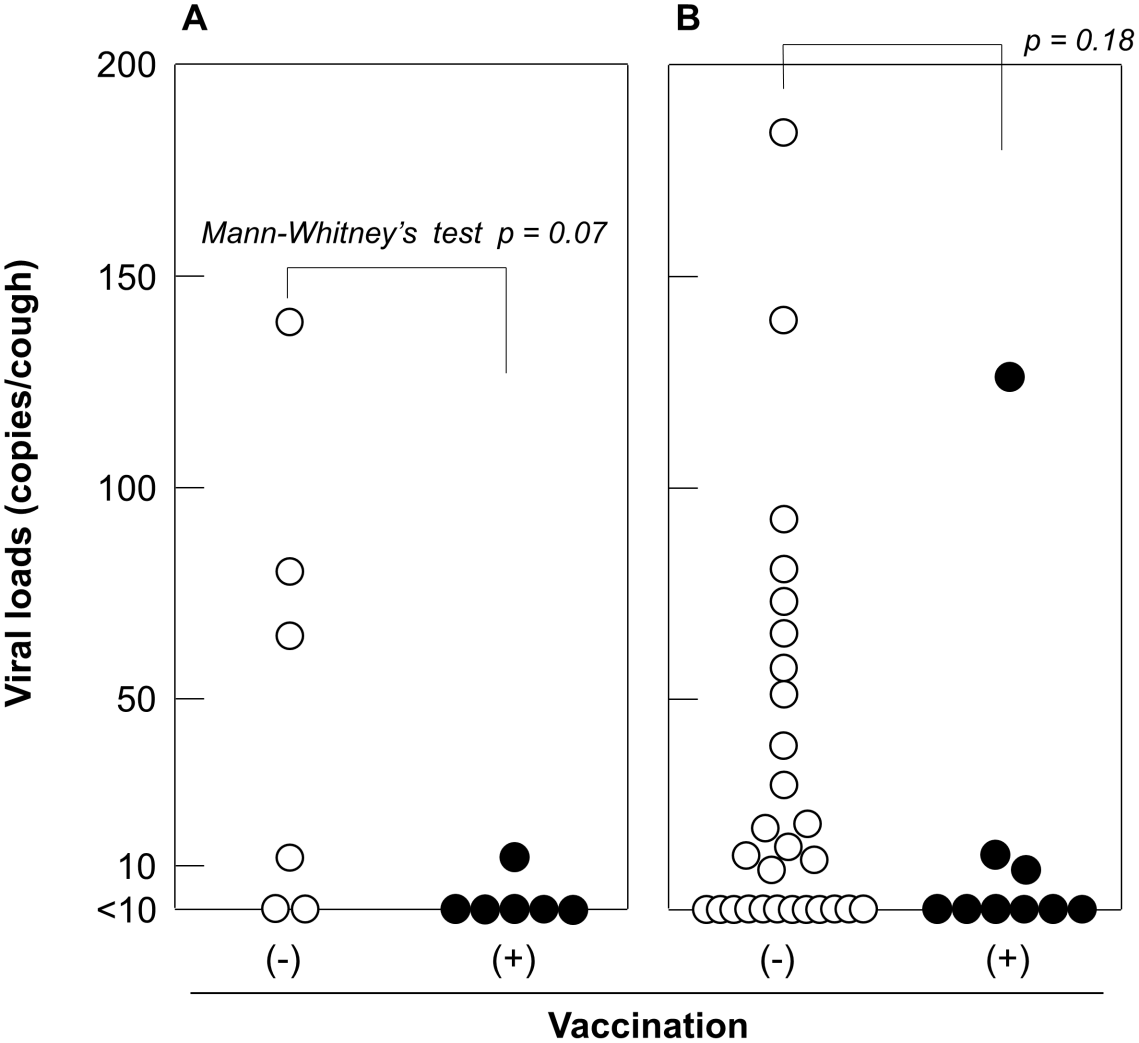
Viral loads in coughs of vaccinated and unvaccinated influenza patients. Viral loads were plotted against vaccination status. Data from days 2 and 3 of cases infected with A(H1) or A(H3) virus are shown together. Viral loads in coughs of antiviral-untreated influenza patients (A) and those of all cases including antiviral-treated cases (B). Open and closed circles represent unvaccinated and vaccinated cases, respectively.

On the other hand, we analyzed cases that did not receive antiviral treatment, by the status of receiving vaccination of seasonal vaccines before onset of seasonal influenza epidemic of subtype H1N1 or H3N2. Viruses were detected in one (17%) of six vaccinated cases and in four (67%) of the six control unvaccinated cases. The viral loads of vaccinated cases were lower than those of the unvaccinated cases (Figure 5 A). These results suggest that vaccination has the potential to suppress viral spread through coughing, although there was only a marginally significant difference in the viral load and virus detection rate results, possibly because of the small sample size (the median viral load  = 12.2 and 72.8 for with and without vaccination; p = 0.07, Mann–Whitney U test). This tendency was similar even when cases that received antiviral treatment were included for analysis (the median viral load  = 12.2 and 44.5, respectively; p = 0.18, Mann–Whitney U test) (Figure 5 B).

### Comparison of viral loads in coughs from patients with and without the symptom of cough

**Figure 6:**
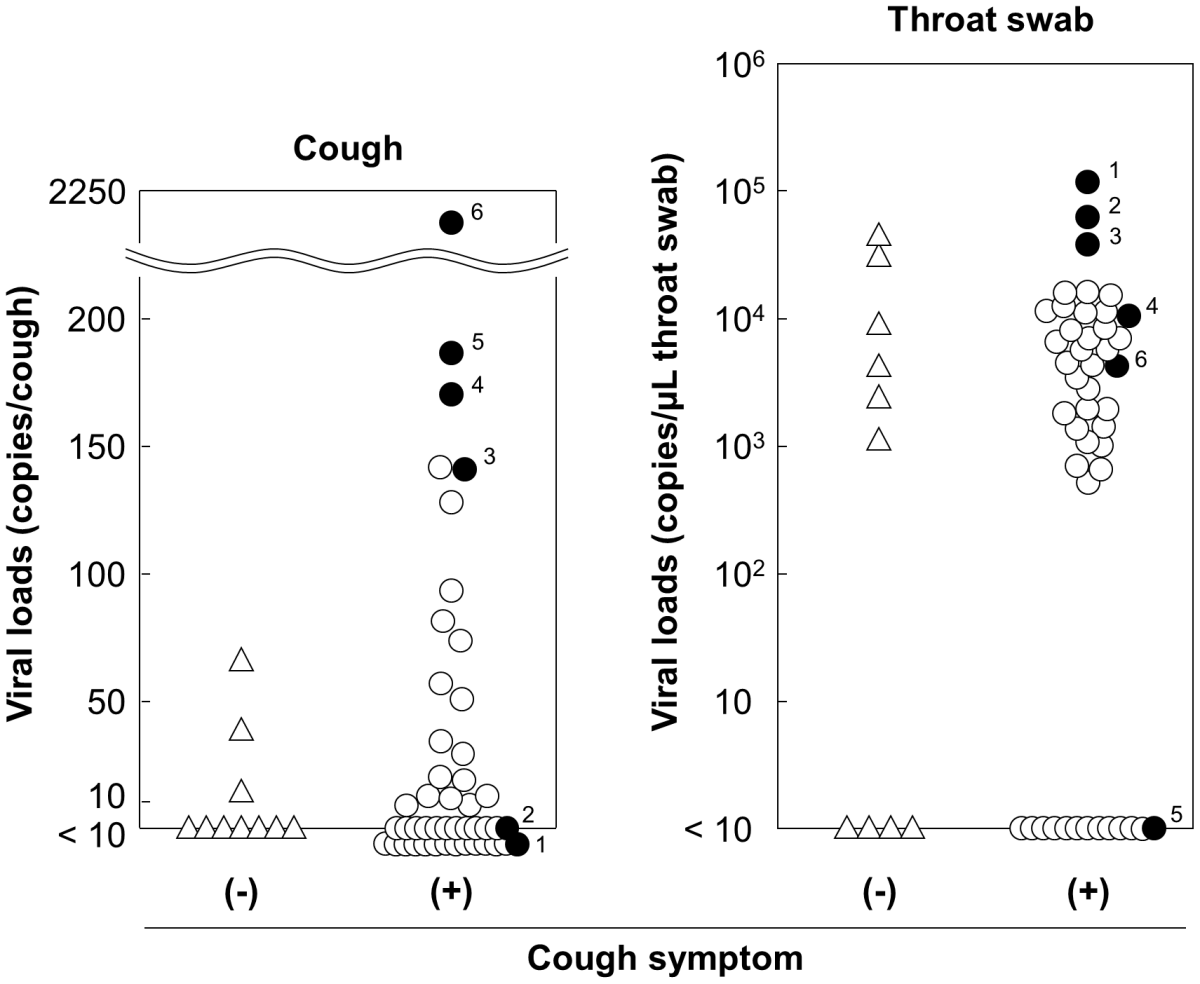
Viral loads of samples from patients with or without cough symptom. Viral loads in cough and throat swab samples from cases with and without a symptom of coughing were compared. The viral load in the throat swab was calculated from the viral load of the transport medium in which the swab fluid was eluted and the volume of the eluted swab fluid, which was tentatively estimated by the increase of the weight of the cotton swab used for sample collection. Circle and triangle represent cases with and without symptoms of coughing. Closed circles indicate cases with higher viral loads among cough or throat swab samples, and identical numbers associated with the closed circles indicate that the data were from the same patient.

In this study, cough samples were collected from patients by voluntary coughing. We thought that it would be important to determine whether the symptom of cough impacted that actual viral loads in a cough; therefore, we considered coughing by non-symptomatic patients, by e.g., due to allergy, physiological stimulations, or causes other than influenza infection. Viral loads in the cough samples collected by voluntary coughing were compared between influenza cases with and without a symptom of coughing using throat swab samples as control (Figure 6). The viral loads in the throat swabs were calculated from the viral load of the transport medium. The approximate quantity of the collected swab fluid was estimated by the increase of the weight of the cotton swab used for sample collection, which was based on the tentative assumption that the specific gravity of the sample was 1.0 and the sample absorbed in the cotton swab was almost completely washed out into the transport medium. The viral load of the cough in patients with the symptom of cough was higher than for those without it (median  = 53.7 and 38.3, respectively). However, the difference was not statistically significant (p = 0.33, Mann–Whitney U test), as was seen with throat swab (median  = 5644 and 6654, respectively; p = 0.79, Mann–Whitney U test). However, interestingly, several patients in the symptomatic group had higher viral loads than in the non-symptomatic group. In addition, we also found that the cases with the highest viral loads in the throat swab (plot numbers 1 and 2) had undetectable levels of viruses in the cough samples; thus, the highest viral load in a cough sample (plot numbers 4–6) did not necessarily correspond with the highest viral load in a throat swab (Figure 6). Hence, our results suggest that the viral load in the cough samples, from at least those cases, might have been affected by the status of the symptom of cough, and do not necessarily directly reflect the status of viral load on the pharyngeal wall.

### Relationships of viral loads in coughs, throat swabs, and oral secretions

**Figure 7:**
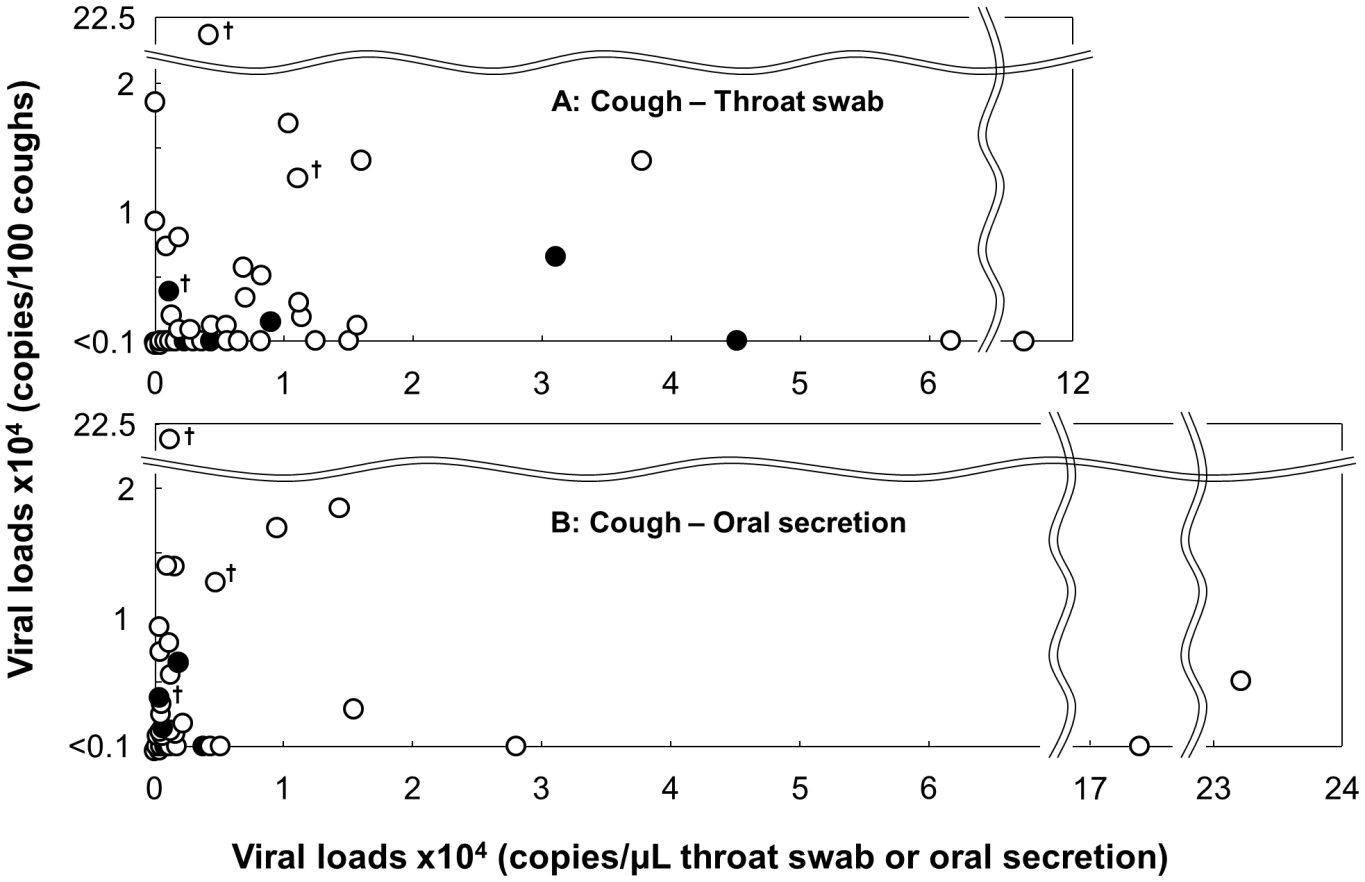
Viral loads of coughs compared to those in throat or oral samples in individual patients. The combined data of viral loads of the cough and throat swab, or cough and oral secretion samples of individual patients were plotted on two-dimensional graphs. The open and closed circles represent cases with and without symptom of coughing, respectively, and daggers indicate cases in which active virus was detected in cough samples using the plaque assay.

In light of these interesting results, we further investigated whether the viral load of cough sample, as collected with our system, reflects those of the pharyngeal walls, oral secretions, or other sources, in relation to from where the virus mainly originated. We plotted combinations of viral loads obtained from cough samples and throat swabs or oral secretions of individual patients in two-dimensional graphs to examine possible correlations (Figure 7 A, B). The Spearman's rank correlation coefficient was also calculated for both combinations. The results showed only weak correlations between both cough samples/throat swabs (r_s_ = 0.355, p = 0.007) and cough samples/oral secretions (r_s_ = 0.528, p = 0.00003). Notably, several cases showed almost no correlations: a high viral load in the throat swab or oral secretion while very low or undetectable in the cough sample, and vice versa (Figure 7 A, B). These results suggest that in some cases from whom the virus was collected from a cough, at least in cases with the highest viral load with very low viral loads in the throat swab or oral samples, the virus in the cough might not have originated from either the throat wall or oral cavity.

## Discussion

The main objective of this study was to develop a simple and portable system to collect and quantify the viral load in a cough with aim to better understand airborne infections. In this regard, we concluded that our system performed well and can be successfully applied to actual influenza patients.

In this system, we employed a gelatin membrane filter to trap viral particles. A review article of aerosol sampling reported that the efficiency of the physical collection of gelatin filters for airborne particles exceeded 96% [17]. Fabian et al. [18] reported that a recovery rate of atomized viral genes using a gelatin filter was 63% of an optimized collection system using the SKC biosampler, but the rate decreased to only 10% for infectious viruses. In the present study, viable viruses were detected in only three samples using a plaque assay. It is possible that the most of trapped viruses became inactivated while trapped in the gelatin filter until the assay was performed.

The overall viral gene recovery rate of our system was estimated at approximately ≥10%, suggesting that the actual viral load in a cough may be 10-fold greater than our actual measured amount, or even greater if we took into account the possible undetected losses during RNA extraction and cDNA synthesis.

In designing the cone shaped, megaphone-like device, we adopted a length of 50 cm to make the airflow velocity between the distal end of the cough and the suction speed of the sampler to ensure optimal air-sampling efficiency. However, we noticed a reflection flow in some actual cases. A stronger cough flow might collide against the inside wall, filter membrane, and/or internal air mass of the device and rebound, or flushed out the internal air mass. However, the result of our preliminary experiments showed that the spill over caused by the outward flow might not affect the result. When a volunteer smoker coughed into the device once in a chamber after one breath of smoking, the smoke particle count adjacent to the outer surface of the device was about 20-fold lower with pumping than without (data not shown). The levels of viral genes measured from coughs in many patients by our system were similar to those measured using the methods of Lindsley et al., who perfectly collected cough samples by asking the patients to blow into a spirometer [8] **,** which may offer supporting evidence to validate the collection rate of our system.

Furthermore, we used universal primers for cDNA synthesis from the viral RNA aimed to quantify the RNA, because we were also preparing to assess influenza B cases during this study period and, therefore, could not use a carrier virus in the ultracentrifuge process. The combined use of specific primers for each viral target and some carrier viruses may improve viral recovery; however, this possibility should be examined in a future study.

Our particle collection method may not sufficiently distinguish large droplets that directly hit the membrane from the small particle droplet nuclei floating inside of the cone, probably because the 50-cm distance from the face to the filter may not long enough to exclude collection of large droplets as well. This possibility was already taken into consideration. Our method was aimed to obtain information regarding the approximate total amount of virus released by coughing.

Apart from the issue of absolute recovery rate, relative comparisons of viral loads among patients may be possible. The highest viral load was 2240 copies/cough among the 56 samples, which was more than 10-fold greater than the average of the virus-positive samples. A possible factor that may play a role in spread of infection is the existence of the so-called “super spreader,” which has been proposed in epidemics of severe acute respiratory syndrome [19] and influenza [20], [21]. Moreover, Lindsley et al. [8] reported a case among a total of 58 suspected influenza cases with a discharge of 355 copies/cough, and Bischoff et al. [9] also reported that 19% of emitters acted as “super emitters,” which was in accordance with the ratio proposed by Lloyd-Smith et al. [20].

Here, we measured the viral loads in cough samples collected from influenza patients. However, there were some limitations to our study regarding the study subjects: most were generally healthy young adults or adolescents, and data from young children, elderly persons, or persons with chronic medical conditions were limited. Second, we could not follow the time course of the viral shedding in the cough among individuals.

We described applications of our methodology to study the effects of vaccination and antiviral treatment, and found that the former may suppress viral release during coughing, but the latter may not, although the former was not yet supported statistically. Regarding antiviral treatment, care should be taken when interpreting the results of this study because all cases treated with oseltamivir were infected with influenza A(H1) virus during the 2008–2009 influenza season, which was considered as resistant to this drug, reflecting the circulating virus at the time of the study [22]. However, the number of subjects included in these analyses was small and may have been insufficient to draw conclusions.

Correlation analyses on the viral loads between the cough and throat swab or oral secretion samples showed that the viral load in a cough may not necessarily reflect those of a throat swab or oral secretion and vice versa, suggesting that the virus in the collected coughs, at least for cases with significant disparities in the viral load level between the two kinds of samples, neither originated from the pharyngeal wall nor the oral cavity, and thus might be mixed with cough air flow at anatomically deeper sites in the respiratory tract than those sites. Regarding the weak correlations between samples, we cannot deny the possibility that in a certain proportion of cases, the virus might have originated from the throat wall or oral cavity as well, or that the virus in the cough may have been trapped in the throat wall and/or oral cavity and we only detected these.

In the present study, we measured the viral load mainly by genetic quantification. However, for optimal infection control, information regarding the activity of virus is more important. We detected active viruses in only 5% of cough samples with moderately high viral titers. This is probably because of the inactivation of the viruses while trapped in the gelatin membrane until plaque titration. We observed that the viruses did not become inactivated if the plaque titration was conducted within a short time after being trapped in the membrane, but they may become inactivated after an extended period with varying degrees of inactivation speed depending on the environmental temperature and humidity (data not shown). This result indicates that our methodology might be insufficient for quantification of viable viruses.

Altogether, our method can be effectively applied for point-of-care detection of influenza viruses from the cough and their quantification. Nonetheless, further large-scale studies are needed to validate our results, which involve several important epidemiological, clinical, and pathophysiological issues.

## Supporting Information

Figure S1 **Size distributions of bio-particles in a human cough and mist generated by a nebulizer.** Comparison of the size distribution of bio-particles from human coughs and mist generated by a nebulizer. The air inside a closed 2 m^3^ chamber was set at about 20% RH and cleaned-up using an air fan unit with a high-efficiency particulate air filter unit. Immediately after healthy adult volunteers coughed voluntarily into the chamber via small window with a flap to seal it, or 2 min after the allantoic fluid of the embryonated chicken egg was atomized in the chamber for 3 s, the particle concentrations were measured using a laser particle-counter (KR-12A; Rion Co., Ltd., Tokyo, Japan) for each particle size range. The data of human cough are presented as absolute particle counts and standard error rang per single cough of the average of five subjects, and the data of the nebulizer are presented as the concentration inside the chamber.
(TIF)

Figure S2 **Viral loads in coughs of influenza patients in different influenza seasons.** The viral load/cough is plotted on the ordinate and the days of illness during sample collection on the abscissa. Epidemic seasons are presented using different symbols: closed circles, open circles, and open triangles represent seasons 2007–2008, 2008–2009, and 2010–2011, respectively.
(TIF)

Figure S3 **Viral loads of coughs of influenza patients by body temperature.** Data of individual cases are plotted on the graph with the viral load in a cough on the ordinate and body temperature at the time of cough sampling on the abscissa. Data of days 2 and 3 were plotted together in the graph. Viral subtypes are presented using different symbols: open circles, closed circles, and open triangles represent infections with A(H1), A(H3), and A(H1N1)pdm09 virus, respectively.
(TIF)

Table S1 **Demographics of virus-detected and undetected cases.**
(TIF)
