# Supplementary figures and images for: Establishment and Clinical Applications of a Portable System for Capturing Influenza Viruses Released through Coughing
Source: PLoS One. 2014 Aug 1;9(8):e103560. doi: 10.1371/journal.pone.0103560 (PMC4118893; doi:10.1371/journal.pone.0103560)

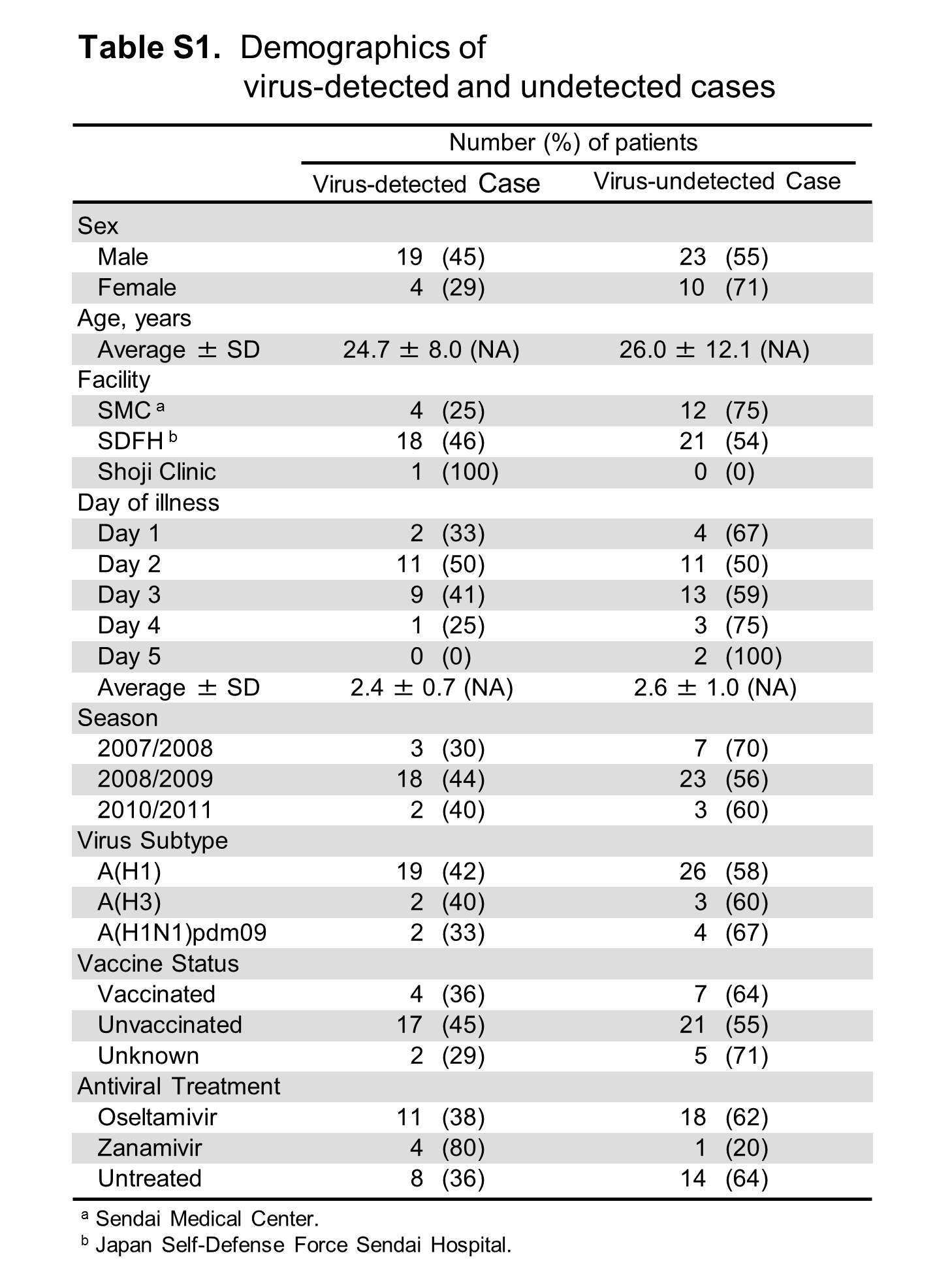

Supplement: Table S1 — Demographics of virus-detected and undetected cases. (TIF) [file pone.0103560.s004.tif]
